# Quantized vs. Full-Precision YOLO Models on Edge Devices: A Performance Benchmark for Real-Time License Plate Detection in Smart Parking Systems

**DOI:** 10.3390/s26165034

**Published:** 2026-08-08

**Authors:** Ervin Burkus, Bence Lestyán, Lehel Dénes-Fazakas, György Eigner

**Affiliations:** 1 Biomatics and Applied Artificial Intelligence Institute, John von Neumann Faculty of Informatics, Obuda University, 1034 Budapest, Hungary; burkus.ervin@uni-obuda.hu (E.B.); eigner.goyrgy@uni-obuda.hu (G.E.); 2 Asura Technologies, 1122 Budapest, Hungary; bence.lestyan@asuratechnologies.com; 3 Physiological Controls Research Center, University Research and Innovation Center, Obuda University, 1034 Budapest, Hungary; 4 MedTech Innovation and Education Center, Obuda University, 1034 Budapest, Hungary

**Keywords:** edge AI, ALPR, object detection, YOLO, quantization, embedded systems, DLPU, inference optimization, computer vision, real-time systems

## Abstract

The deployment of deep learning-based vision systems on edge devices introduces a complex trade-off between computational efficiency and detection accuracy. In this work, we investigate this trade-off in the context of a multi-stage Automatic License Plate Recognition (ALPR) pipeline, evaluated in two heterogeneous edge execution environments: a general-purpose Raspberry Pi 5 single-board computer and the ARTPEC-8 system-on-chip integrated into an Axis smart camera, where neural network inference is accelerated by the on-chip DLPU. All experiments were performed using pre-recorded images loaded from the file system; neither the Axis camera sensor nor a live video stream was used. This study evaluates the impact of model architecture, numerical precision, and input resolution on both inference latency and detection performance. YOLOv5- and YOLOv8-based models were analyzed under multiple quantization schemes (FP32, FP16, dynamic, and INT8), while a cross-platform benchmark was conducted to assess the benefits and limitations of hardware acceleration. The results show that dedicated accelerators provide significant latency reduction at higher resolutions; however, this advantage is accompanied by reduced flexibility and increased sensitivity to quantization effects. In contrast, CPU-based execution enables the use of more recent and quantization-robust model architectures, which can partially compensate for the lack of hardware acceleration when combined with resolution scaling. Furthermore, the analysis hig ights the importance of hybrid-resolution processing in multi-stage pipelines, where different stages can operate at different input resolutions to balance accuracy and performance. The findings demonstrate that optimal system design requires a joint consideration of hardware characteristics, model architecture, and quantization strategy, rather than relying on a single optimization dimension. The presented results provide practical insights for the design of efficient and robust edge-based ALPR systems, with direct implications for real-world industrial deployments.

## 1. Introduction

Deep learning-based computer vision has recently shifted from centralized server-based processing toward edge devices [1]. This shift is mainly driven by the need for real-time performance, reduced bandwidth usage, and improved data privacy. At the same time, edge devices such as smart cameras operate under strict limitations in terms of computational power, energy consumption, and thermal constraints. These limitations make the deployment of complex multi-stage vision pipelines a challenging task.

To make such systems feasible on edge hardware, dedicated accelerators (e.g., NPUs, DLPUs) and model optimization techniques (such as quantization and pruning) are commonly used [2,3]. These approaches aim to reduce computational cost while maintaining acceptable accuracy.

However, numerical precision and input resolution introduce coupled rather than independent design trade-offs. Reducing numerical precision decreases model size and memory traffic and may accelerate inference on hardware with native low-precision support. At the same time, quantization can introduce calibration and rounding errors that affect classification confidence and bounding-box localization, particularly for small or weakly represented objects. Increasing the input resolution preserves more spatial detail and generally improves the detection of small vehicles and license plates; however, it also increases the number of processed pixels approximately quadratically, resulting in higher latency, memory usage, and computational demand.

The optimal configuration is therefore strongly hardware-dependent. FP16 or INT8 conversion does not necessarily provide the same acceleration on a general-purpose ARM CPU as on a dedicated neural processing unit, while accelerator-based execution may impose restrictions regarding supported operators, model structures, calibration procedures, and numerical formats. Consequently, model architecture, quantization precision, input resolution, and execution hardware must be optimized jointly rather than considered as independent design parameters.

It should be emphasized that the present experiments evaluate the on-device processing environment rather than the image-acquisition subsystem. The Axis camera sensor was not used during the measurements. Instead, identical pre-recorded images were loaded from the file system on both execution environments. Consequently, camera exposure, image-sensor characteristics, image signal processing, video compression, network transmission, and live stream acquisition were excluded from the reported latency and accuracy measurements.

This research was conducted in collaboration with Asura Technologies and was motivated by a practical requirement arising in smart parking and vehicle-access applications. Conventional ALPR deployments commonly rely on an external x86-based processing unit connected to one or more cameras. Such a configuration increases hardware cost, installation complexity, power consumption, physical space requirements, and maintenance effort.

The industrial objective is to determine whether the computationally demanding neural network components of an ALPR system can be transferred to the processing hardware already integrated into an Axis smart camera. A successful on-camera implementation could reduce dependence on external computing infrastructure, simplify installation, and enable more scalable deployments at parking entrances and controlled-access locations. The present study provides a stage-level benchmark supporting this engineering decision, although it does not represent validation of a commercially released product.

**Figure 1:**
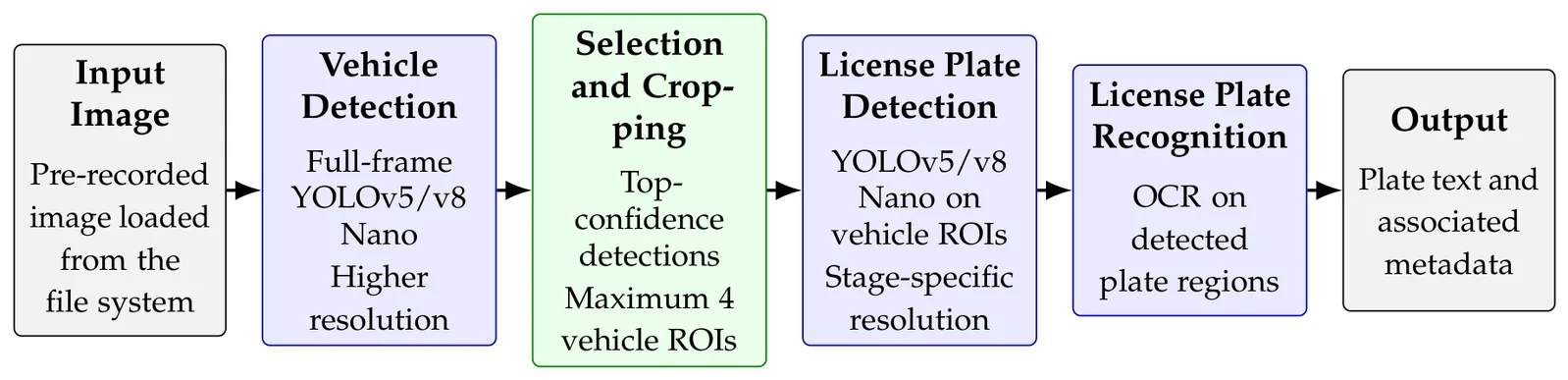
Resource-aware cascaded ALPR processing pipeline. Vehicle detection is performed on the complete input image, after which only the four highest-confidence vehicle regions are forwarded to the license plate detector. The two detection stages can operate at different input resolutions according to their spatial and computational requirements.

The proposed system uses a cascaded three-stage ALPR processing pipeline, referred to in this work as the “Triple-DNN” pipeline (see Figure 1). It consists of the following main stages:

1. **Vehicle Detection:** Vehicles are detected in the input image, and the most confident detections are selected.
2. **License Plate Detection:** For each detected vehicle, a cropped region is extracted from the original image, and the license plate is localized within this region.
3. **License Plate Recognition (OCR):** The text on the detected license plate is recognized using either deep learning-based or classical OCR methods.

The term “Triple-DNN” does not denote a newly proposed YOLO backbone or detection head. Instead, it refers to the resource-aware orchestration of the three consecutive processing stages. Its main characteristics are introduced at the system-design level.

First, the cascaded organization progressively reduces the search space. License plate detection is performed only within the vehicle regions identified by the first detector, rather than over the complete input image. Second, only the four highest-confidence vehicle detections are forwarded to the subsequent stage, establishing an upper bound on the downstream computational workload. Third, the modular organization enables the individual stages to be configured with different input resolutions. Full-frame vehicle detection may require a higher resolution to preserve information about small and distant objects, whereas the license plate detector processes cropped vehicle regions in which the plate occupies a larger proportion of the input image. This allows a lower resolution to be considered for the subsequent stage without applying the same resolution reduction to the complete scene. Finally, the modular design permits the models used at the individual stages to be adapted to the numerical precision and execution constraints of the target hardware.

Accordingly, the contribution of the Triple-DNN configuration is a hardware-aware and resolution-aware deployment strategy for a cascaded ALPR pipeline, together with a stage-level analysis of its computational requirements. It is not presented as a new standalone neural network architecture.

For detection, we use lightweight YOLO-based models that are suitable for edge deployment [4,5,6].

A key engineering question is whether the neural network components of the proposed pipeline can be executed within the computational constraints of the target edge hardware. The present study addresses this question through a cross-platform benchmark of the first and computationally significant stage: full-frame vehicle detection. DLPU-accelerated inference is compared with general-purpose ARM CPU execution, and the effects of numerical precision and input resolution are evaluated in terms of latency and detection accuracy.

The reported measurements do not constitute an end-to-end evaluation of the complete ALPR pipeline. In particular, the downstream license plate detector and OCR stage are not included in the reported latency and accuracy results. The benchmark instead provides stage-level evidence that can support the later selection of hardware, model architecture, numerical precision, and input resolution for the complete system.

Overall, the goal of this work is to provide a practical stage-level assessment of the computationally significant vehicle-detection component of an edge-based ALPR pipeline, hig ighting the trade-offs between latency and detection accuracy [7].

To address these challenges, this work presents a systematic evaluation of a multi-stage edge AI pipeline for automatic license plate recognition across heterogeneous embedded platforms.

**The main contributions of this work are summarized as follows:**

- A cross-platform benchmark comparing CPU-based and DLPU-accelerated inference in a multi-stage ALPR pipeline.
- A detailed analysis of the impact of numerical precision on detection robustness, including distribution-level evaluation beyond average metrics.
- An investigation of the trade-offs between model architecture and hardware constraints in edge deployments.
- The identification of a hybrid-resolution processing strategy that improves latency while maintaining detection performance.
- A resource-aware cascaded ALPR pipeline that reduces the downstream search space through vehicle-region cropping and bounds the computational workload by forwarding only the highest-confidence detections.
- A stage-specific hybrid-resolution strategy in which full-frame vehicle detection and cropped license plate detection are executed at resolutions selected according to their different spatial and computational requirements.

## 2. Related Work

The deployment of deep learning models on edge devices has introduced fundamental changes in the design of embedded systems. In this section, we review the dominant hardware architectures, memory constraints, energy efficiency aspects, and benchmarking methodologies.

### 2.1. Hardware Paradigms and Memory Hierarchy

The literature typically distinguishes three main approaches for running inference on edge devices.

The first is the CPU-centric approach, where general-purpose ARM processors are used. As discussed in [8], Cortex-A series processors (e.g., those used in Raspberry Pi devices) provide a flexible solution due to their general instruction set. Software-level optimizations, such as ARM NEON SIMD extensions, can improve performance, but the achievable computing density remains limited compared to specialized hardware.

The second category consists of dedicated tensor accelerators (e.g., NPU, DLPU, TPU), which are ASIC-based solutions optimized for low-precision matrix operations. A well-known limitation of such accelerators is their strong dependence on on-chip memory capacity. As shown in [9], these devices often exhibit a “bi-modal” performance behavior: as long as the model fits into the limited on-chip SRAM (typically around a few megabytes), performance is high; however, once external memory access (DRAM) is required, latency increases significantly. This constraint has a direct impact on model design choices in industrial edge systems.

The third paradigm is based on embedded GPUs, such as the NVIDIA Jetson family. According to [10], these platforms perform well for hig y parallel workloads, but their higher power consumption and thermal requirements limit their applicability in low-power edge camera systems. More recent studies, such as [11], confirm that GPUs remain advantageous for flexibility, but are less suitable for strict power-constrained deployments.

### 2.2. Energy Efficiency and Thermal Constraints

In edge AI applications, energy efficiency is often as important as latency. Measurements reported in [8] indicate that dedicated accelerators can consume as little as 4% of the energy required by CPU-based solutions for certain tasks. In practice, this translates to a significant difference in power budgets: for example, a Google Coral TPU typically operates around 2 W, while an NVIDIA Jetson Nano requires approximately 10 W, and more advanced platforms such as Xavier may exceed 30 W [10].

Power consumption is closely related to thermal behavior. As observed in [12], sustained workloads can trigger dynamic voltage and frequency scaling (DVFS), leading to fluctuations in inference latency. This is particularly critical in real-time applications such as ALPR, where consistent frame rates are required.

### 2.3. Benchmarking Methodologies and Metrics

Comparing edge AI platforms is inherently challenging due to their heterogeneity. As hig ighted in [13], metrics such as the Power-Delay Product (PDP) and “inferences per Joule” provide a more comprehensive view of system efficiency. The PDP, defined as the product of power consumption and latency, captures the trade-off between speed and energy usage.

To ensure fair comparisons, standardized benchmarking frameworks have been proposed. One example is DeepEdgeBench [14], which unifies model conversion and evaluation procedures across different platforms. Other efforts, such as MLPerf Tiny [15], aim to establish widely accepted benchmarks specifically for resource-constrained edge devices.

### 2.4. Coupled Effects of Quantization and Input Resolution

Quantization and resolution scaling are among the most widely used techniques for adapting deep neural networks to resource-constrained edge platforms. Post-training quantization reduces numerical precision, model size, and memory bandwidth requirements, potentially improving inference latency and energy efficiency [2,3]. Nevertheless, the resulting speedup depends on whether the target hardware and inference runtime provide native support for the selected numerical format. Unsupported operators, precision conversions, or fallback execution may substantially reduce the expected performance benefit [8,16].

For object detection, quantization may influence both classification and localization. Small changes in activation and weight representations can affect confidence scores and bounding-box regression, even when average detection metrics remain nearly unchanged. Consequently, quantized models may retain competitive mean performance while exhibiting increased variability or a greater number of low-quality detections. Calibration-set representativeness, quantization granularity, and the use of post-training or quantization-aware training are therefore important practical considerations.

Input resolution introduces a related trade-off. Higher resolutions preserve fine-grained spatial information and facilitate the detection of small objects, which is particularly important for license plates occupying only a limited portion of the original scene. However, increasing the side length of a square input from $r_{1}$ to $r_{2}$ increases the number of processed pixels by a factor of ${\left(r_{2}/r_{1}\right)}^{2}$. This results in increased latency, memory traffic, and energy consumption. Conversely, aggressive resolution reduction may remove relevant spatial details before they can be processed by the detector.

The effects of quantization and resolution are therefore interdependent. The loss of spatial information at low resolution may amplify quantization-related degradation, whereas higher resolutions may offset part of the accuracy loss at the cost of substantially increased computation. Existing edge AI benchmarks frequently investigate hardware acceleration, numerical precision, or resolution scaling separately [14,15]. Comparatively fewer studies evaluate their combined influence in cascaded ALPR systems, where errors in an upstream vehicle detector propagate to the subsequent license plate detection and recognition stages. This coupled optimization problem represents the main motivation for the present study.

### 2.5. Hardware–Software Co-Optimization

Inference performance is influenced not only by hardware, but also by the software stack. As demonstrated in [16], the choice of runtime environment (e.g., TensorFlow Lite vs. ARM NN) can result in runtime differences of up to 86%.

In addition, compatibility between model operators and hardware support plays a crucial role. As noted in [8], newer architectures such as MobileNetV3 may underperform compared to older variants if certain operators are not efficiently supported by the target hardware. Similar observations have been reported in [17], where architectural improvements depend heavily on hardware-aware optimization.

These findings motivate the design choices in this work. In particular, we adopt YOLO-based architectures and utilize the native Axis LAROD API to ensure efficient execution and maximum hardware utilization on the target platform.

To the best of our knowledge, no prior work has jointly analyzed hardware acceleration, quantization, and resolution effects in a multi-stage ALPR pipeline.

## 3. Materials and Methods

Experimental validation was carried out in two heterogeneous edge execution environments with fundamentally different architectures. The primary test environment was an Axis smart camera containing the ARTPEC-8 system-on-chip. ARTPEC-8 is not a standalone embedded computing platform comparable to the Raspberry Pi 5; rather, it is the integrated processing unit of the Axis smart camera and contains the DLPU used for neural network inference. The evaluated configuration reflects an industrial deployment scenario in which ALPR processing is intended to operate directly within an Axis smart camera rather than on an external computing unit.

As a reference platform, a Raspberry Pi 5 single-board computer was used. This device serves two purposes. First, it provides a baseline for evaluating the performance of the Triple-DNN architecture on general-purpose ARM cores. Second, it allows for testing model variants with different numerical precisions, which is not possible on the Axis platform. While the ARTPEC-8 DLPU supports only 8-bit fixed-point (INT8) inference, the Raspberry Pi enables direct comparison between FP32, FP16, and INT8 models. This makes it possible to estimate the impact of quantization and to assess the portability of the system across platforms.

### 3.1. Hardware Platforms

The two platforms differ significantly in terms of CPU architecture and computational capability. The Raspberry Pi 5 is based on the Broadcom BCM2712 SoC, which integrates a quad-core ARM Cortex-A76 processor [18]. The Cortex-A76 cores provide approximately 10.7 DMIPS/MHz, offering relatively high compute performance for a general-purpose embedded platform. Since no dedicated AI accelerator is available, neural network inference is executed on the CPU, using ARM NEON (Advanced SIMD) instructions via the TensorFlow 2.13 Lite XNNPACK delegate.

In contrast, the Axis ARTPEC-8 SoC contains a dual-core ARM Cortex-A53 processor (approximately 2.3 DMIPS/MHz) [19], and relies on a dedicated DLPU (Deep Learning Processing Unit) for neural network inference.

Although a newer ARTPEC-9 platform is already available, this work focuses on ARTPEC-8, as it is still widely deployed in current industrial systems. Using this platform ensures that the results remain relevant for existing real-world applications.

The main hardware characteristics of the evaluated platforms are summarized in Table 1.

### 3.2. Software Architecture

To ensure a fair comparison, the processing logic and measurement methodology were kept consistent across both platforms. Although each device uses a platform-specific software stack (Axis LAROD vs. TensorFlow Lite with XNNPACK), the functional steps of the pipeline (preprocessing, tensor preparation, and postprocessing) follow the same structure.

The application is implemented as a native processing pipeline, with direct control over memory allocation and tensor operations. Basic image processing steps—such as resizing, color conversion, and letterboxing—were implemented manually at a low level. This approach minimizes overhead and allows for better control over memory layout and data alignment, which is particularly important on resource-constrained devices.

A custom benchmarking framework was developed to measure the individual stages of the Triple-DNN pipeline, with a focus on the detection stage. The framework provides millisecond-level profiling of preprocessing, inference, and postprocessing steps. On the Axis platform, models were converted into device-specific INT8 format to enable execution on the DLPU.

The main software characteristics are summarized in Table 2.

To ensure reproducibility and eliminate external factors, all measurements were performed using image inputs loaded from the file system. This guarantees identical input data across platforms and avoids variability caused by video compression or ISP (Image Signal Processor) settings. As a result, the evaluation focuses strictly on computational performance and recognition accuracy.

### 3.3. Model Architecture

The choice of the object detection model is a key design decision, especially in edge environments where memory and computational resources are limited. In this work, we use models from the YOLO (You Only Look Once) family [22], which are widely adopted in real-time embedded vision systems due to their favorable speed–accuracy trade-off.

Alternative architectures exist. Two-stage detectors such as Faster R-CNN typically provide higher accuracy, but their computational cost makes them impractical for edge deployment. On the other hand, lightweight single-stage models such as SSD [23] and MobileNet-based detectors [24] are designed for efficiency, but in many cases offer lower detection performance. Comparative studies suggest that YOLO-based models provide a good balance between inference speed and accuracy for real-time applications [22,25].

The evaluated vehicle-detection models were initialized using weights pretrained on the COCO (Common Objects in Context) dataset [26]. COCO contains 80 object categories, including vehicle classes relevant to the first stage of the proposed pipeline. However, COCO does not contain a license plate class. Therefore, the COCO-based models evaluated in this study correspond exclusively to the full-frame vehicle detector and should not be interpreted as license plate detection models.

In this study, we focus on the YOLOv5 [27] and YOLOv8 [6] model families. YOLOv5 is used as a stable baseline, representing the traditional anchor-based detection approach. In contrast, YOLOv8 introduces an anchor-free design and a decoupled detection head, which separates classification and localization tasks. This structural change is one of the main differences between the two generations and has practical implications for both performance and model optimization.

From a deployment perspective, model size is a critical factor. Both YOLOv5 and YOLOv8 are available in multiple variants (nano, small, medium, large, and extra-large). Due to the constraints of the target edge platform, only the smallest “nano” variants are considered in this work. The ARTPEC-8 platform has limited memory, and the Triple-DNN pipeline requires multiple models to be loaded simultaneously. Under these conditions, larger models are not feasible if real-time performance is required.

On the Axis ARTPEC-8 platform, the standard YOLOv5n architecture is used. This choice is primarily driven by compatibility with the vendor-provided optimization tools [28] and the LAROD API, which ensure stable deployment on the DLPU.

On the Raspberry Pi reference platform, both YOLOv8n and a modified YOLOv5 variant (YOLOv5nu) are evaluated. The latter incorporates a decoupled detection head, making it structurally closer to YOLOv8. This modification has been reported to improve robustness during fixed-point (INT8) conversion, which is particularly relevant for edge deployment.

Overall, the selected models allow us to compare different design choices—anchor-based vs. anchor-free, coupled vs. decoupled heads—under the same application constraints, while keeping the computational requirements within the limits of the target hardware.

The selection of YOLOv5 and YOLOv8 was primarily determined by deployment compatibility, reproducibility, and the constraints of the target industrial hardware rather than by an intention to benchmark every available YOLO generation. On the Axis ARTPEC-8 platform, YOLOv5n was selected because it is supported by the vendor-provided conversion and optimization workflow and can be executed reliably through the LAROD API. On the Raspberry Pi 5, YOLOv5nu and YOLOv8n were included because both can be exported to TensorFlow Lite and executed using the same CPU-based inference environment.

More recent generations, such as YOLOv11 and YOLOv12, were not included because they were not part of the validated ARTPEC-8 deployment workflow used in this study. Evaluating these models only on the Raspberry Pi would extend the architectural comparison but would not provide a corresponding cross-platform configuration on the target smart-camera hardware. Their evaluation is therefore left for future work, once a reproducible conversion and execution path is available for the industrial target platform.

### 3.4. Model Quantization and Conversion

Adapting the models to the target hardware requires careful selection of numerical precision and model format, as these directly affect inference performance. In this work, two separate conversion pipelines were used, reflecting the architectural constraints of the evaluated platforms.

On the Raspberry Pi platform, the Ultralytics framework was used to export PyTorch 2.9.1-based models to TensorFlow Lite format. This pipeline supports both standard YOLOv5 models and newer variants with a decoupled detection head (YOLOv5nu). In this structure, classification and localization are handled by separate branches, which can improve optimization behavior and, in some cases, lead to more stable performance after quantization.

For evaluation, four model variants with different numerical precision were generated:

- 32-bit floating point (FP32);
- 16-bit floating point (FP16);
- Dynamically quantized model (dynamic range quantization);
- Fully quantized INT8 model using a calibration dataset.

This setup allows for a direct comparison between different precision levels and provides insight into the trade-offs between accuracy and runtime performance.

For the Axis ARTPEC-8 platform, model conversion followed the vendor-provided workflow [28]. This process is based on a modified YOLOv5 architecture that is specifically adapted for the DLPU. In this case, the standard YOLOv5n model with a coupled detection head was used, as it is fully supported by the available optimization tools and the LAROD API.

During conversion, the models were quantized to INT8 using a per-tensor scheme, in accordance with the requirements of the DLPU [29]. The corresponding dequantization parameters were integrated into the runtime environment based on the model metadata, ensuring correct interpretation of the inference outputs.

Overall, the two conversion pipelines reflect different design priorities: flexibility and precision control on the Raspberry Pi, and hardware compatibility and efficiency on the Axis platform.

Evaluating object detection models on embedded platforms requires considering both runtime performance and detection quality. Model compression and quantization improve efficiency, but may affect accuracy. Therefore, the applied methodology focuses on quantifying the trade-off between speed and detection performance under realistic deployment conditions.

### 3.5. Experimental Scope and Evaluated Detection Stage

The Triple-DNN pipeline shown in Figure 1 represents the target architecture of the industrial ALPR system. However, the experimental benchmark reported in this study evaluates only the first stage of this pipeline: full-frame vehicle detection. The UA-DETRAC dataset provides vehicle-level bounding-box annotations but does not provide license plate annotations or character-level recognition labels. Consequently, it can be used to evaluate the vehicle detector, but not the downstream license plate detector or OCR stage.

All inference-latency, F1-score, and IoU results presented in this manuscript therefore refer to a single execution of the full-frame vehicle detector. They do not represent an aggregated evaluation of the two detection networks and do not constitute an end-to-end evaluation of the complete ALPR pipeline. Similarly, the FP32, FP16, dynamic-range, and INT8 labels refer to the numerical precision of the evaluated vehicle-detection model only.

The license plate detection and OCR stages are included in the system diagram to describe the intended deployment architecture and to motivate the stage-specific resolution strategy. Their training datasets, recognition accuracy, and end-to-end runtime are outside the scope of the present benchmark. A complete pipeline evaluation will require a separate dataset containing license plate bounding boxes and transcription annotations, together with stage-specific accuracy and cumulative latency measurements.

### 3.6. Dataset and Preprocessing

Experiments were conducted on the UA-DETRAC dataset [30], which contains real-world traffic scenes captured from fixed camera viewpoints. This setup closely matches the intended application scenario of the evaluated edge-based ALPR system.

The YOLO models used in this work are pretrained on the COCO dataset [26], which defines 80 object categories. In contrast, UA-DETRAC contains four vehicle-related classes: *Car*, *Bus*, *Van*, and *Others*. To ensure compatibility, a mapping was applied between the two label spaces:

- COCO Car → DETRAC Car;
- COCO Bus → DETRAC Bus;
- COCO Truck → DETRAC Van/Others.

The original DETRAC images have a resolution of 960×540 pixels (aspect ratio 16:9), while the YOLO models expect square inputs. Direct resizing would distort object geometry, therefore a letterbox approach was used. Each image was placed into a 960×960 frame with zero-padding, preserving the original aspect ratio.

To reduce redundancy and ensure variability in the evaluation set, uniform temporal subsampling was applied. Every 50th frame was selected from each video sequence, resulting in a dataset of approximately 1200 images. This subset captures a range of environmental conditions, including different lighting and weather scenarios, while avoiding near-duplicate samples.

### 3.7. Evaluation Metrics

Detection performance was assessed using commonly applied object detection metrics. The overlap between a predicted bounding box, $B_{\mathrm{pred}}$, and the corresponding ground-truth bounding box, $B_{\mathrm{gt}}$, was quantified using the Intersection over Union (IoU):

(1) $$\mathrm{IoU}=\frac{\left|B_{\mathrm{pred}}\cap B_{\mathrm{gt}}\right|}{\left|B_{\mathrm{pred}}\cup B_{\mathrm{gt}}\right|},$$

A predicted bounding box was regarded as a correct detection when the IoU defined in Equation (1) exceeded the predefined threshold τ=0.5. Based on this criterion, the detection outcomes were classified as true positives (TP), false positives (FP), and false negatives (FN).

Precision represents the proportion of predicted detections that were correctly identified and was calculated as follows:

(2) $$P=\frac{\mathrm{TP}}{\mathrm{TP}+\mathrm{FP}},$$

whereas recall indicates the proportion of ground-truth objects that were successfully detected and was calculated as follows:

(3) $$R=\frac{\mathrm{TP}}{\mathrm{TP}+\mathrm{FN}}.$$

The F1 score was additionally used to provide a single measure that balances precision and recall:

(4) $$F_{1}=2\frac{P\cdot R}{P+R}.$$

To ensure a consistent and fair comparison, the same inference thresholds were applied across all evaluated platforms:

- Confidence threshold: 0.25;
- NMS threshold: 0.45;
- IoU threshold: τ=0.5.

### 3.8. Runtime Measurement

The object detection pipeline consists of three stages: preprocessing, inference, and postprocessing. Preprocessing includes image loading, resizing, color conversion, and normalization. Inference corresponds to executing the neural network on the target hardware, while postprocessing includes decoding the network outputs and applying non-maximum suppression.

In this study, the analysis focuses on inference time, as it represents the dominant computational cost. Preprocessing and postprocessing overhead is relatively small and depends on implementation details that are not directly comparable across platforms.

To ensure consistency, all inputs were loaded from the file system, providing identical data for both platforms. This avoids variability introduced by video pipelines or ISP configurations and allows for a direct comparison of computational performance.

On the Raspberry Pi platform, measurements were carried out under controlled conditions. The CPU governor was set to *performance* mode, and the operating frequency was fixed at 2.4 GHz. Thermal conditions were monitored throughout the experiments to ensure stable operation without throttling. This setup minimizes runtime variability and improves reproducibility.

## 4. Results

In this section, we compare the performance of the evaluated models across the two platforms. On the Raspberry Pi 5, YOLOv5nu and YOLOv8n were tested using multiple numerical precisions (FP32, FP16, Dynamic, INT8). On the Axis ARTPEC-8 platform, the analysis focuses on the YOLOv5n model using the hardware-supported INT8 configuration. The results are presented for three input resolutions. Unless explicitly stated otherwise, every result in this section refers exclusively to the first-stage full-frame vehicle detector. The results are not aggregated across vehicle detection, license plate detection, and OCR.

The evaluation is divided into two parts: inference latency and detection accuracy.

### 4.1. Inference Latency

**Figure 2:**
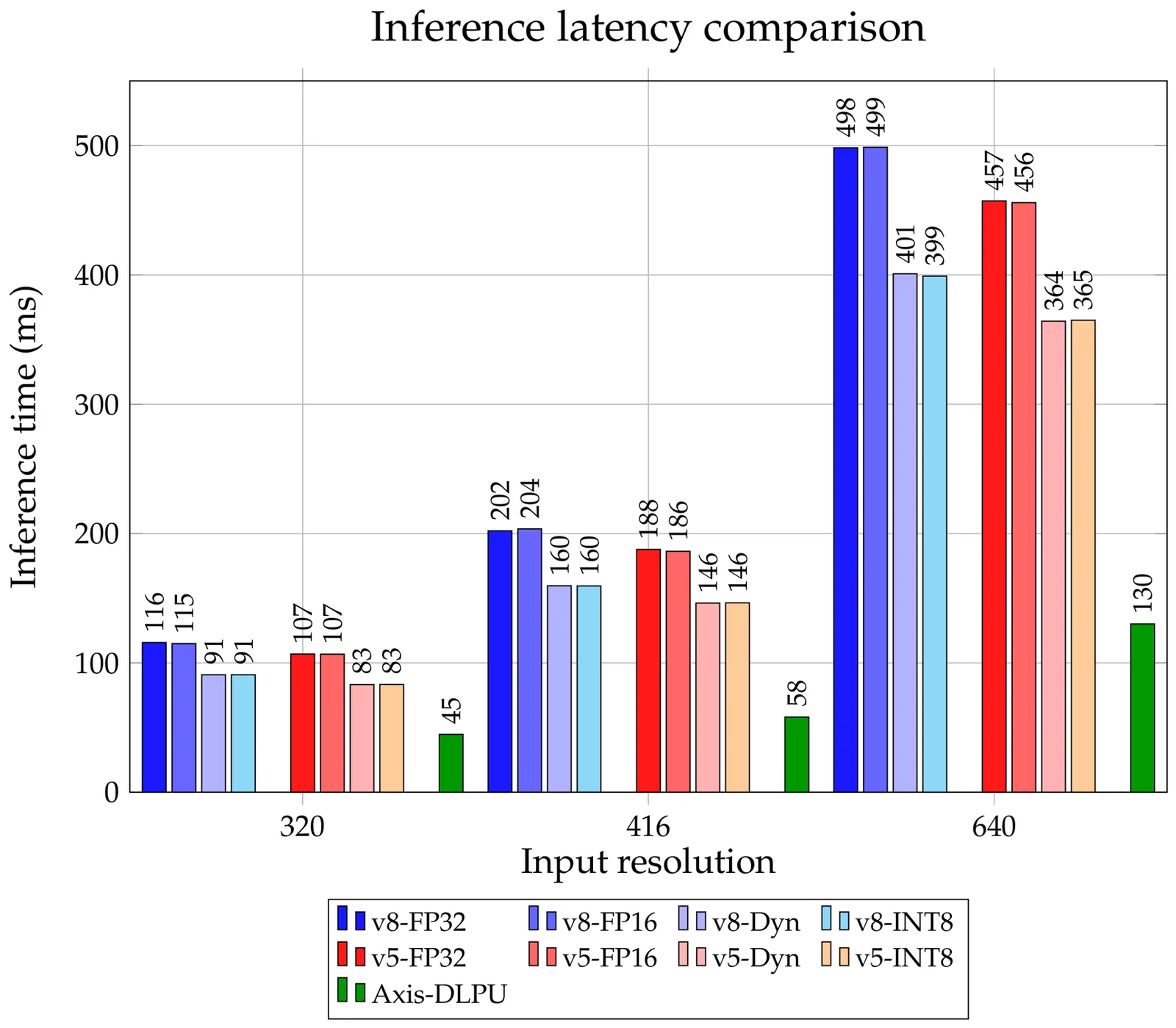
Measured inference latency of the evaluated object detection models at 320 × 320, 416 × 416, and 640 × 640 input resolutions. The Raspberry Pi 5 results include YOLOv8n and YOLOv5nu models executed using FP32, FP16, dynamic-range quantization, and full INT8 quantization. The Axis result corresponds to the YOLOv5n model executed in INT8 precision on the ARTPEC-8 DLPU. Values above the bars indicate inference latency in milliseconds; lower values represent faster execution.

The measured inference times are shown in Figure 2. The two platforms exhibit clearly different performance characteristics.

**Model comparison (YOLOv5nu vs. YOLOv8n):** On the Raspberry Pi, YOLOv5nu consistently achieves lower latency than YOLOv8n. At 640×640 resolution, the INT8 version of YOLOv5nu runs in 364–365 ms, which is approximately 9% faster than YOLOv8n (399 ms). This difference can be attributed to architectural complexity, indicating that the modified YOLOv5 variant remains a competitive choice for edge deployment.

**Floating-point precision (FP32 vs. FP16):** No significant difference was observed between FP32 and FP16 execution. At 640×640, YOLOv8n runs in 498 ms (FP32) and 499 ms (FP16). This confirms that the tested ARM architecture does not provide a practical advantage for FP16 computations in the TensorFlow Lite environment.

**Quantized models (Dynamic vs. INT8):** Dynamic range quantization and full INT8 quantization show nearly identical performance. Both provide approximately 20% speedup compared to floating-point models. From a practical standpoint, dynamic quantization is easier to apply, as it does not require a calibration dataset, while still achieving similar performance.

**Cross-platform deployment-stack comparison:** At 640×640 resolution, the Axis ARTPEC-8 configuration completes inference in approximately 130 ms, whereas the fastest Raspberry Pi 5 configuration requires approximately 364 ms. This corresponds to an observed configuration-level speed difference of approximately 2.8×. However, this result should not be interpreted as an isolated comparison of the two hardware processors. The evaluated environments use different YOLOv5 variants, model-conversion pipelines, inference runtimes, and acceleration mechanisms. Consequently, the measured difference reflects the combined effect of hardware, model architecture, numerical representation, and software implementation.

**Resolution scaling:** The relative advantage of the DLPU decreases at lower resolutions. At 416×416, the speedup is ∼2.5× (58 ms vs. 146 ms), while at 320×320 it drops to ∼1.8× (45 ms vs. 83 ms). This trend indicates that hardware acceleration becomes more beneficial as computational complexity increases.

This observation is consistent with [9], where a similar “crossover” behavior is reported. At lower workloads, fixed overheads (e.g., initialization and data movement) account for a larger fraction of the total runtime.

### 4.2. Detection Accuracy

**Figure 3:**
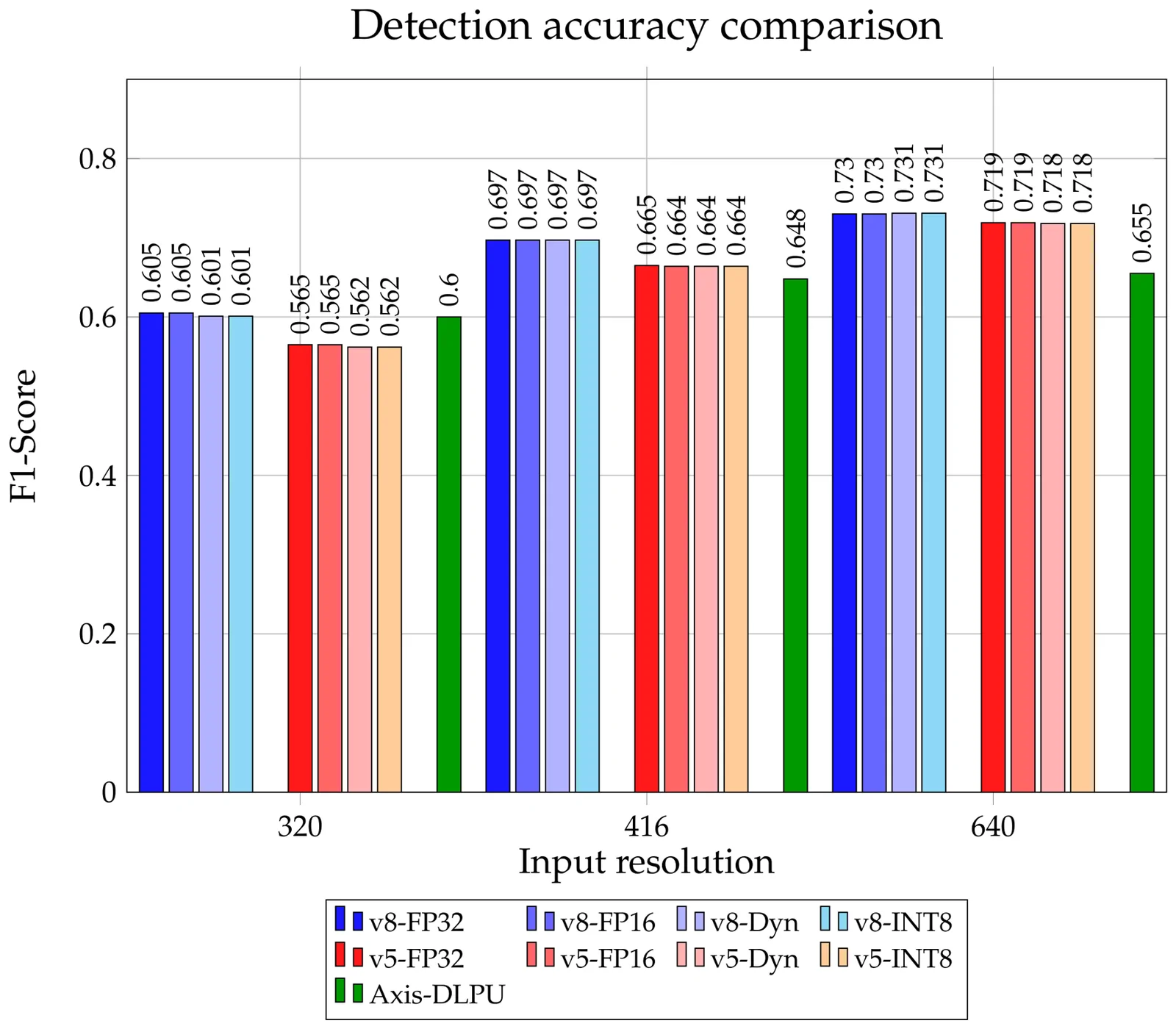
Detection performance of the evaluated model configurations at 320 × 320, 416 × 416, and 640 × 640 input resolutions, expressed as F1-score at an IoU threshold of 0.5. Raspberry Pi 5 results are reported for YOLOv8n and YOLOv5nu using FP32, FP16, dynamic-range, and INT8 precision, whereas the Axis result represents YOLOv5n INT8 inference on the ARTPEC-8 DLPU. Higher F1-scores indicate better overall detection performance.

Detection performance is summarized in Figure 3.

**Effect of quantization:** On the Raspberry Pi, reducing precision from FP32 to INT8 has minimal impact on F1-score (Δ<0.005). This indicates that quantization does not significantly degrade detection quality for the tested models in a CPU environment.

**Model comparison:** YOLOv8n consistently outperforms YOLOv5nu in terms of accuracy. At 640×640, YOLOv8n achieves an F1-score of 0.730, compared to 0.719 for YOLOv5nu.

**Axis ARTPEC-8 configuration:** At 640×640 resolution, the Axis YOLOv5n INT8 configuration achieves an F1-score of 0.655, whereas the Raspberry Pi YOLOv8n configurations achieve approximately 0.730. This difference represents a comparison between complete deployment configurations rather than an isolated hardware or quantization effect. Since the two environments differ in model architecture, detection head, conversion procedure, inference runtime, and numerical implementation, the present experiments do not permit the observed accuracy difference to be attributed to a single factor.

**Resolution impact:** Higher input resolution significantly improves detection performance. For example, YOLOv5nu increases from 0.565 (320) to 0.719 (640). This hig ights the trade-off between accuracy and latency in the system design.

### 4.3. Qualitative Analysis

**Figure 4:**
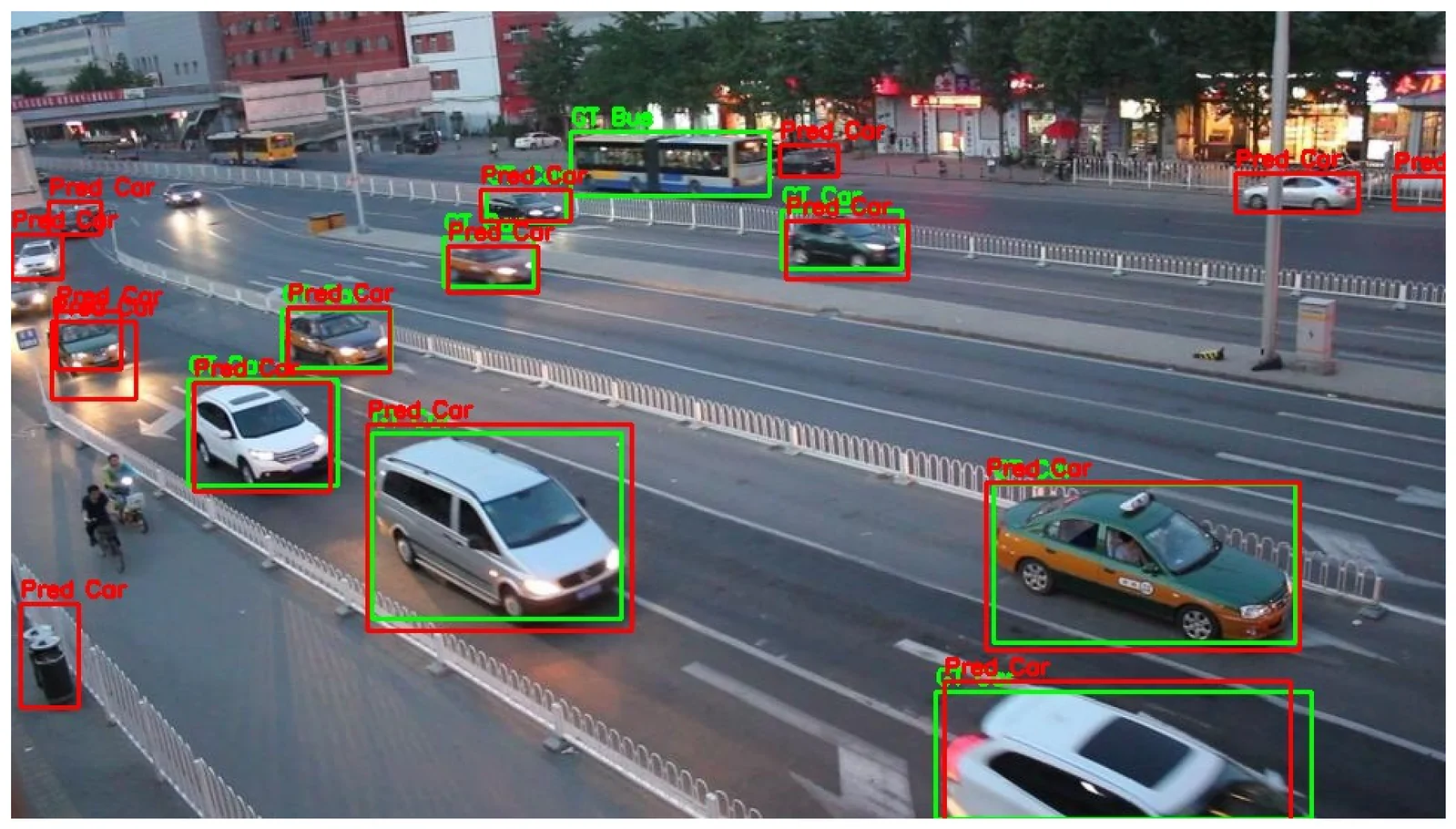
Qualitative example of vehicle detection on an image from the UA-DETRAC evaluation set. Green bounding boxes represent the ground-truth annotations, while red bounding boxes represent model predictions. Red boxes without a corresponding ground-truth annotation are treated as false positives during evaluation, although some of them correspond to visually identifiable vehicles that are missing from the dataset annotations.

A qualitative example is shown in Figure 4. In most cases, predicted bounding boxes closely match the ground truth annotations. However, several small distant vehicles are detected by the model but missing from the annotations. These are counted as false positives during evaluation, which suggests that the reported F1-scores may underestimate the actual detection capability.

At the same time, typical failure cases can be observed. These include missed detections (false negatives) and misclassifications caused by visual similarity between objects.

### 4.4. Comprehensive Analysis of All Model Configurations

**Figure 5:**
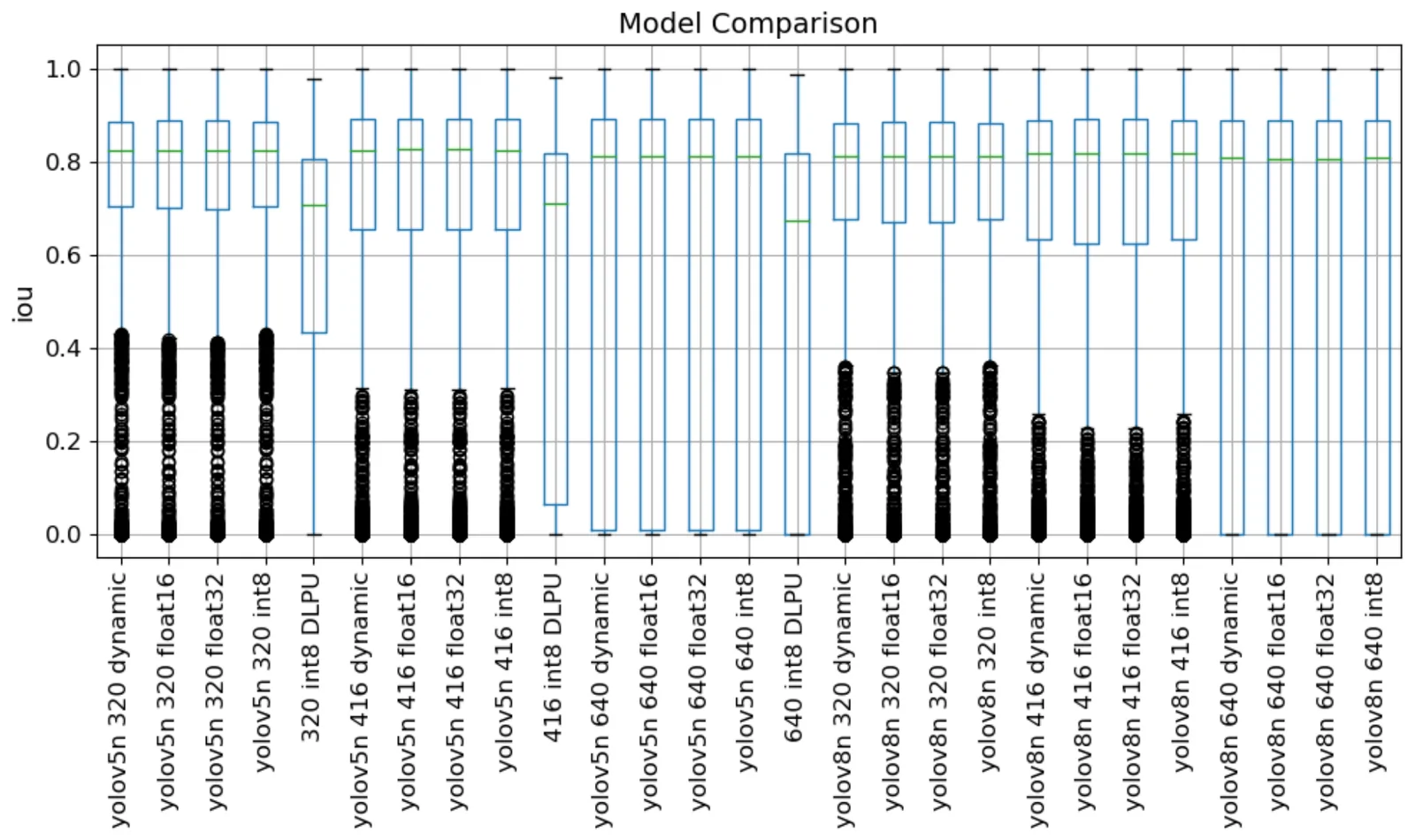
Distribution of Intersection over Union (IoU) values for all evaluated detector configurations, grouped by model family, input resolution, numerical precision, and execution platform. Raspberry Pi 5 configurations include YOLOv5nu and YOLOv8n models using FP32, FP16, dynamic-range, and INT8 precision, whereas the DLPU configurations correspond to YOLOv5n INT8 execution on the Axis ARTPEC-8. The center line of each box represents the median, the box indicates the interquartile range, and the individual markers show observations outside the main distribution.

The distributional characteristics of the Intersection over Union (IoU) metric across all evaluated model configurations reveal systematic performance variations as a function of architectural generation, input resolution, and numerical precision (see Figure 5). The corresponding descriptive statistics are summarized in Table 3. Rather than relying solely on central tendency measures, the present analysis emphasizes dispersion, robustness, and the prevalence of low-IoU outliers, which are critical indicators for deployment in real-world environments.

A clear dependency on input resolution can be observed. Models operating at 320×320 resolution exhibit lower median IoU values and increased interquartile ranges compared to higher-resolution counterparts. This effect is particularly pronounced in quantized implementations. Increasing the input resolution to 416×416 results in improved median IoU and reduced variability, indicating enhanced spatial localization capabilities. The 640×640 configurations consistently demonstrate the highest median IoU values and narrower interquartile spreads, suggesting improved boundary delineation and better detection of small or structurally complex objects. These findings confirm the expected trade-off between computational cost and spatial precision, with higher-resolution inputs enabling more accurate feature representation and localization.

Regarding numerical precision, the comparison between float32 and float16 implementations shows negligible degradation in IoU performance. The median values and dispersion metrics remain nearly identical across most resolutions and architectures, indicating that reduced precision arithmetic does not significantly compromise detection quality. This observation is particularly relevant for hardware-constrained systems, where half-precision computation offers substantial memory and throughput advantages without measurable accuracy loss.

In contrast, int8-quantized models exhibit a broader distribution of IoU values and a noticeably higher density of low-IoU outliers. The degradation is more evident at lower resolutions, where quantization-induced representation constraints compound spatial information loss. Although the central tendency of int8 models may remain competitive in certain configurations, the increased variance and outlier frequency indicate reduced robustness. This suggests that quantization-aware training or post-training calibration strategies are essential when deploying such models in safety-critical or precision-sensitive applications.

Architectural differences between YOLOv5 and YOLOv8 further contribute to the observed performance trends. Across nearly all resolutions and precision settings, YOLOv8-based configurations achieve higher median IoU values and exhibit reduced dispersion compared to YOLOv5 variants. The tighter interquartile ranges imply improved generalization stability and more consistent localization performance. These improvements can be attributed to architectural refinements in feature aggregation, detection head optimization, and training strategies introduced in the newer generation. The advantage becomes increasingly evident at higher resolutions, where the architectural capacity can more effectively exploit richer spatial information.

The presence and distribution of outliers provide additional insight into model robustness. A significant number of near-zero IoU values appear primarily in lower-resolution and quantized configurations, indicating complete or near-complete detection failures in specific samples. Such failures may arise from missed detections, severe bounding box misalignment, or difficulties in detecting small-scale targets. High-resolution floating-point models show a reduced frequency of extreme outliers, suggesting greater stability under challenging visual conditions.

Overall, the analysis demonstrates that optimal performance is achieved through a balanced combination of architectural advancement, sufficient spatial resolution, and appropriate numerical precision. While higher resolutions consistently improve localization accuracy, the marginal gains must be weighed against increased computational demands. Float16 precision offers a favorable compromise between efficiency and accuracy, whereas int8 quantization introduces measurable robustness degradation unless carefully optimized. Importantly, evaluating only average IoU would obscure critical distributional characteristics; dispersion metrics and outlier behavior are essential for a comprehensive assessment of practical reliability and deployment readiness.

### 4.5. Comparative Analysis of YOLOv5n and YOLOv8n Architectures

**Figure 6:**
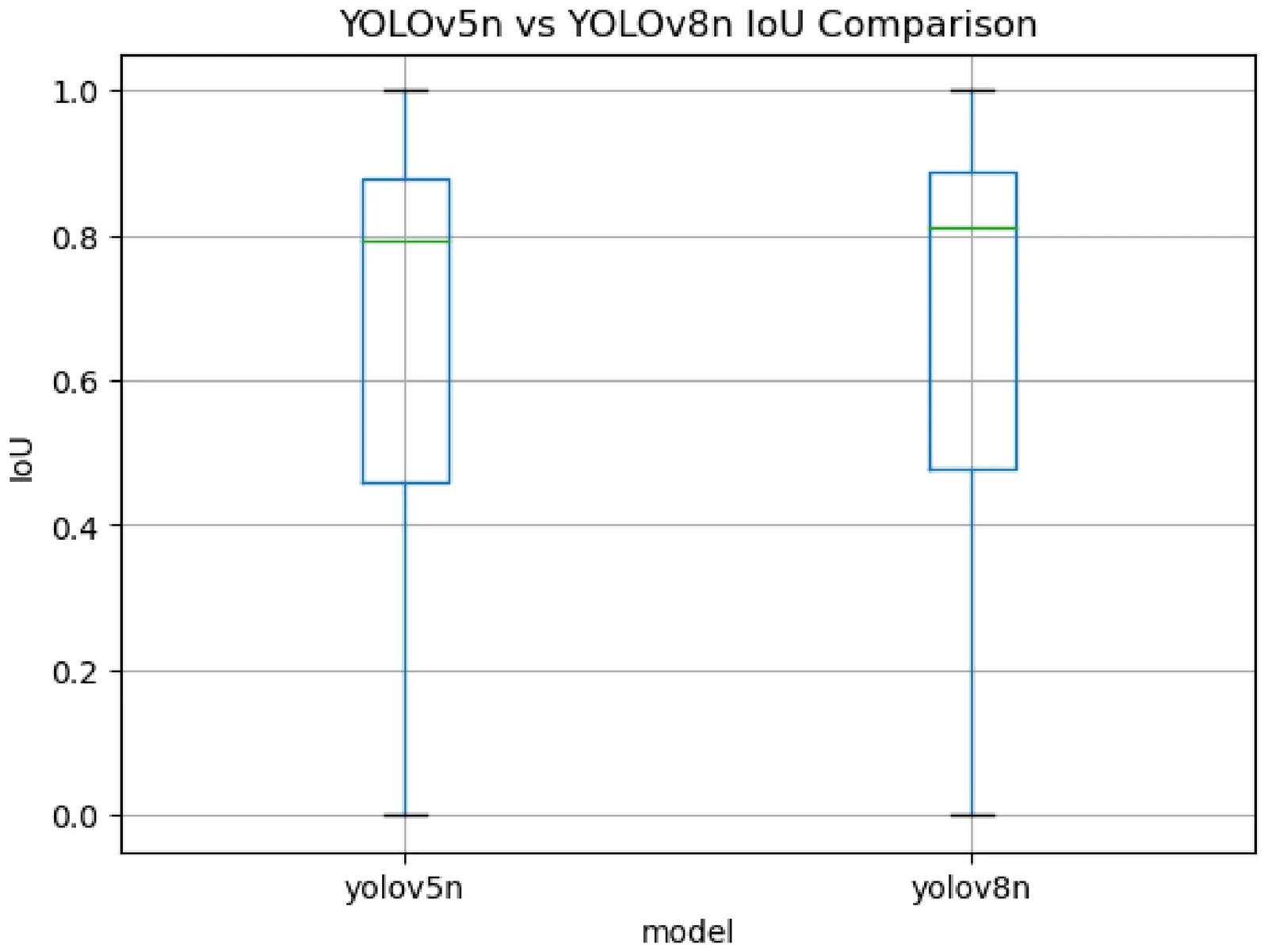
Aggregated comparison of the IoU distributions obtained with YOLOv5-based and YOLOv8n detectors across the evaluated resolutions and numerical precision formats. The YOLOv5-based group combines the Raspberry Pi 5 YOLOv5nu configurations and the Axis ARTPEC-8 YOLOv5n INT8 configurations, whereas the YOLOv8n group contains the Raspberry Pi 5 results. The boxplots summarize the median, interquartile range, overall dispersion, and extreme IoU observations.

The comparative evaluation of YOLOv5n and YOLOv8n models, based on the distribution of Intersection over Union (IoU) scores, provides insight into architectural efficiency, localization stability, and robustness characteristics (see Figure 6). The corresponding descriptive statistical measures are summarized in Table 4. The analysis focuses not only on central tendency but also on dispersion properties and extreme-value behavior, which are critical for assessing deployment reliability.

The median IoU of YOLOv8n is marginally higher than that of YOLOv5n, indicating a consistent improvement in localization accuracy. Although both models reach upper whisker values approaching 1.0, suggesting that near-perfect detections are achievable in optimal cases, the distributional structure reveals subtle but meaningful differences. YOLOv8n exhibits a slightly tighter interquartile range, implying more stable detection performance across heterogeneous samples. In contrast, YOLOv5n demonstrates a broader spread, reflecting higher variability in bounding box alignment quality.

The lower quartile values of both models indicate the presence of challenging samples that substantially reduce IoU performance. However, YOLOv8n maintains a comparatively elevated first quartile, suggesting improved robustness under adverse conditions such as small object size, occlusion, or complex background textures. This shift in the lower distribution boundary is particularly relevant for real-world applications where worst-case behavior often determines system reliability.

Both architectures show whiskers extending toward zero, indicating occasional severe localization failures. Nevertheless, the density and dispersion of low-IoU instances appear moderately reduced in YOLOv8n. This observation aligns with architectural enhancements introduced in the YOLOv8 generation, including refined feature aggregation strategies and improved detection head optimization, which contribute to more consistent spatial predictions.

From a statistical stability perspective, YOLOv8n demonstrates reduced variance while maintaining or slightly improving median performance. This combination suggests better generalization capacity rather than isolated performance gains on specific samples. The improvement is therefore not merely reflected in central tendency but also in the contraction of the variability envelope.

Overall, the distributional comparison indicates that YOLOv8n achieves superior localization robustness and marginally higher median accuracy relative to YOLOv5n. While both models remain competitive and capable of high IoU performance, the tighter distribution and improved lower-bound behavior of YOLOv8n provide evidence of architectural maturation. Importantly, the evaluation underscores that performance assessment should extend beyond mean IoU values to incorporate dispersion and outlier characteristics, as these factors critically influence operational reliability in practical deployment scenarios.

### 4.6. Resolution-Centric Performance Analysis

**Figure 7:**
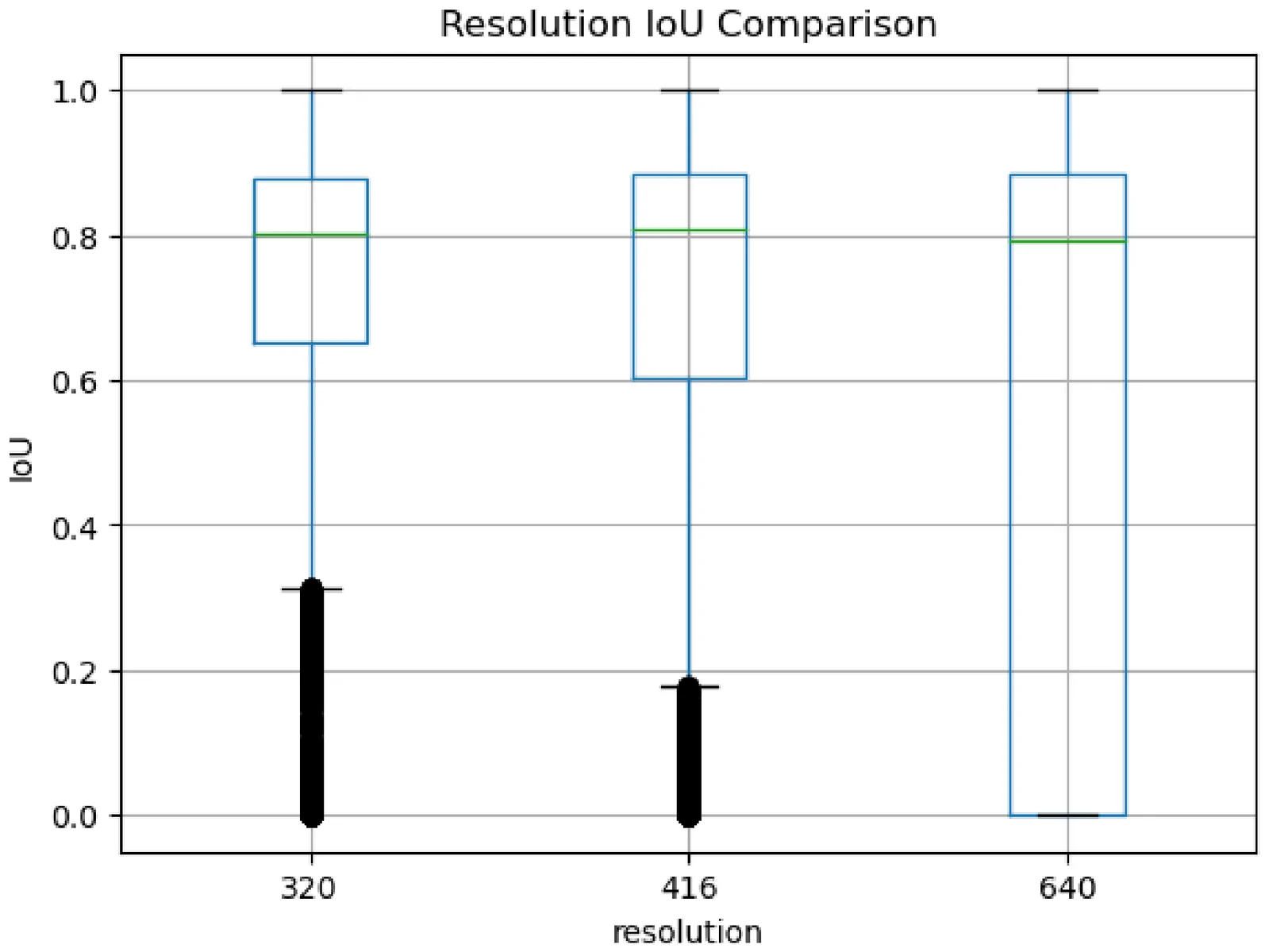
Aggregated IoU distributions at 320 × 320, 416 × 416, and 640 × 640 input resolutions. Each group combines the evaluated model architectures, numerical precision formats, and execution platforms operating at the corresponding resolution. The boxplots illustrate how input resolution affects the median localization accuracy, interquartile variability, and the occurrence of low-IoU cases.

The impact of input resolution on localization performance was investigated by analyzing the distribution of Intersection over Union (IoU) scores across the evaluated resolutions (320, 416, and 640 pixels). The distributional characteristics are illustrated in Figure 7, while the corresponding descriptive statistical indicators are summarized in Table 5. The analysis emphasizes not only median performance but also dispersion properties and extreme-value behavior, which are critical for understanding robustness and deployment stability.

A consistent structural trend can be observed across resolutions. The 320-pixel configuration demonstrates competitive median IoU values; however, it exhibits a considerable number of low-IoU outliers. This indicates that while the model is capable of accurate detections under favorable conditions, it is more prone to substantial localization degradation in challenging samples. The relatively wider spread of lower-bound values suggests sensitivity to small object scales and fine-grained spatial details, which may be underrepresented at reduced input resolution.

Increasing the resolution to 416 pixels results in a modest upward shift in the median IoU and a contraction of the interquartile range. The lower whisker remains extended, but the density of extreme low-IoU cases is reduced compared to the 320 configuration. This behavior indicates improved spatial feature representation and more stable bounding box regression, reflecting a favorable balance between computational cost and localization precision.

The 640-pixel configuration presents a distinct distributional structure. While the median IoU remains comparable to or slightly higher than that of the lower resolutions, the interquartile range expands significantly due to a bifurcation in performance distribution. A substantial concentration of near-zero IoU values is observed, alongside a cluster of high-IoU detections approaching 1.0. This bimodal tendency suggests that the highest resolution enhances peak localization accuracy but may introduce instability under certain conditions, potentially due to increased model sensitivity, overfitting effects, or resolution-dependent training dynamics.

From a robustness perspective, the 416-pixel configuration appears to provide the most stable compromise, combining strong median performance with relatively controlled variance. The 320-pixel setting offers computational efficiency but at the cost of increased susceptibility to localization failures. Conversely, the 640-pixel resolution maximizes spatial detail representation yet demonstrates greater dispersion and extreme-case variability, which may affect predictability in operational environments.

Overall, the findings confirm that input resolution is a dominant factor influencing IoU distribution characteristics. Higher resolutions do not uniformly guarantee improved robustness; instead, they modify the balance between central accuracy and variance. Consequently, resolution selection should be guided by application-specific constraints, considering both computational resources and tolerance to performance variability. Importantly, the distributional analysis hig ights that median IoU alone is insufficient to characterize resolution effects, as dispersion and outlier behavior substantially influence real-world reliability.

### 4.7. Precision-Oriented IoU Performance Analysis

**Figure 8:**
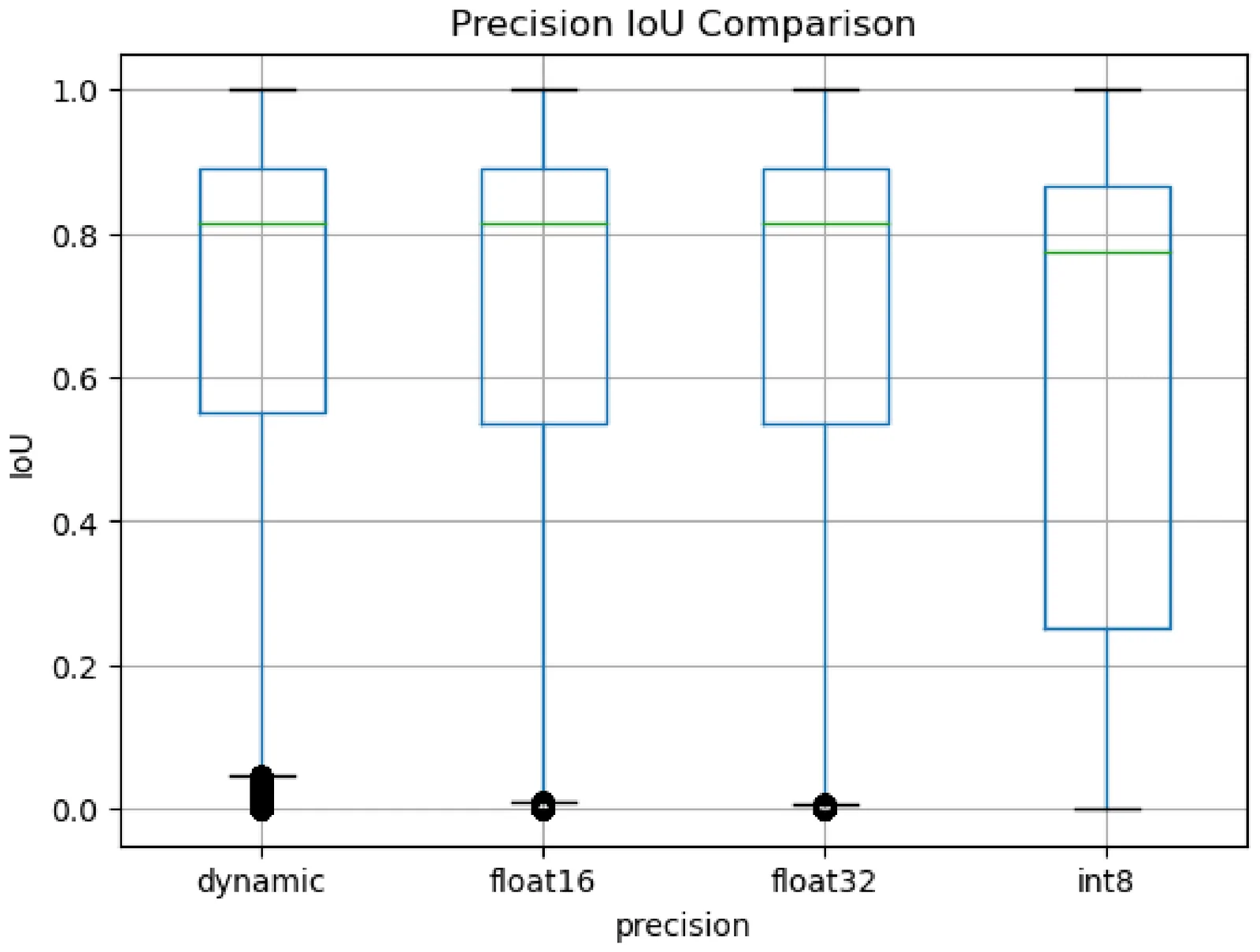
Aggregated IoU distributions for dynamic-range, FP16, FP32, and INT8 model representations across the evaluated architectures and input resolutions. The dynamic-range, FP16, and FP32 groups contain Raspberry Pi 5 results, whereas the INT8 group contains both Raspberry Pi 5 and Axis ARTPEC-8 DLPU configurations. The boxplots show the median, interquartile range, overall dispersion, and extreme localization outcomes associated with each numerical format.

The influence of numerical precision on localization performance was examined by analyzing the distribution of Intersection over Union (IoU) values across dynamic, float16, float32, and int8 implementations. The distributional behavior is illustrated in Figure 8, while the corresponding descriptive statistics are reported in Table 6. The evaluation emphasizes central tendency, dispersion characteristics, and the prevalence of extreme-value cases to assess robustness under different arithmetic representations.

The dynamic, float16, and float32 configurations exhibit hig y similar median IoU values, all centered around the upper performance range. Their interquartile ranges substantially overlap, indicating that reduced-precision floating-point arithmetic (float16) does not introduce statistically significant degradation compared to full precision (float32). The dynamic configuration behaves consistently with the floating-point variants, suggesting stable inference behavior when adaptive precision strategies are employed.

A closer examination of the lower distribution boundary reveals that dynamic, float16, and float32 models maintain comparable first-quartile values and whisker extents. Although low-IoU outliers are present in all cases, their density and spread remain relatively constrained. This indicates that floating-point implementations preserve stable bounding box regression and classification confidence even under challenging visual conditions.

In contrast, the int8-quantized configuration demonstrates a markedly different distributional profile. While the upper whisker still reaches near-perfect IoU values, indicating that high-quality detections are achievable, the median IoU is slightly reduced and the interquartile range is considerably wider. More notably, a substantial expansion of the lower-bound region is observed, with a pronounced concentration of near-zero IoU values. This suggests increased sensitivity to quantization-induced representation constraints, particularly affecting fine-grained localization and small-object detection.

The broader dispersion and heavier lower tail of the int8 distribution indicate reduced robustness relative to floating-point implementations. Quantization compresses weight and activation ranges, potentially amplifying rounding effects and diminishing regression precision. Although quantized inference provides substantial computational and memory advantages, these benefits are accompanied by measurable variability in localization performance.

From a deployment perspective, float16 emerges as an efficient compromise, offering computational acceleration with negligible impact on IoU distribution characteristics. Full float32 precision provides marginal stability advantages but may not justify the additional resource requirements in constrained environments. The int8 configuration, while computationally attractive for edge devices and DPU-based systems, requires careful calibration or quantization-aware training to mitigate the observed degradation in lower-bound performance.

Overall, the results demonstrate that numerical precision significantly influences IoU distribution structure, particularly in terms of variance and extreme-case behavior. While central tendency metrics remain relatively stable across floating-point formats, quantization introduces a shift in robustness characteristics that must be explicitly considered during model selection and hardware deployment planning. Importantly, distributional analysis reveals nuances that would remain obscured if evaluation were restricted to mean IoU values alone.

### 4.8. DLPU Versus Non-DLPU Inference Performance Analysis

**Figure 9:**
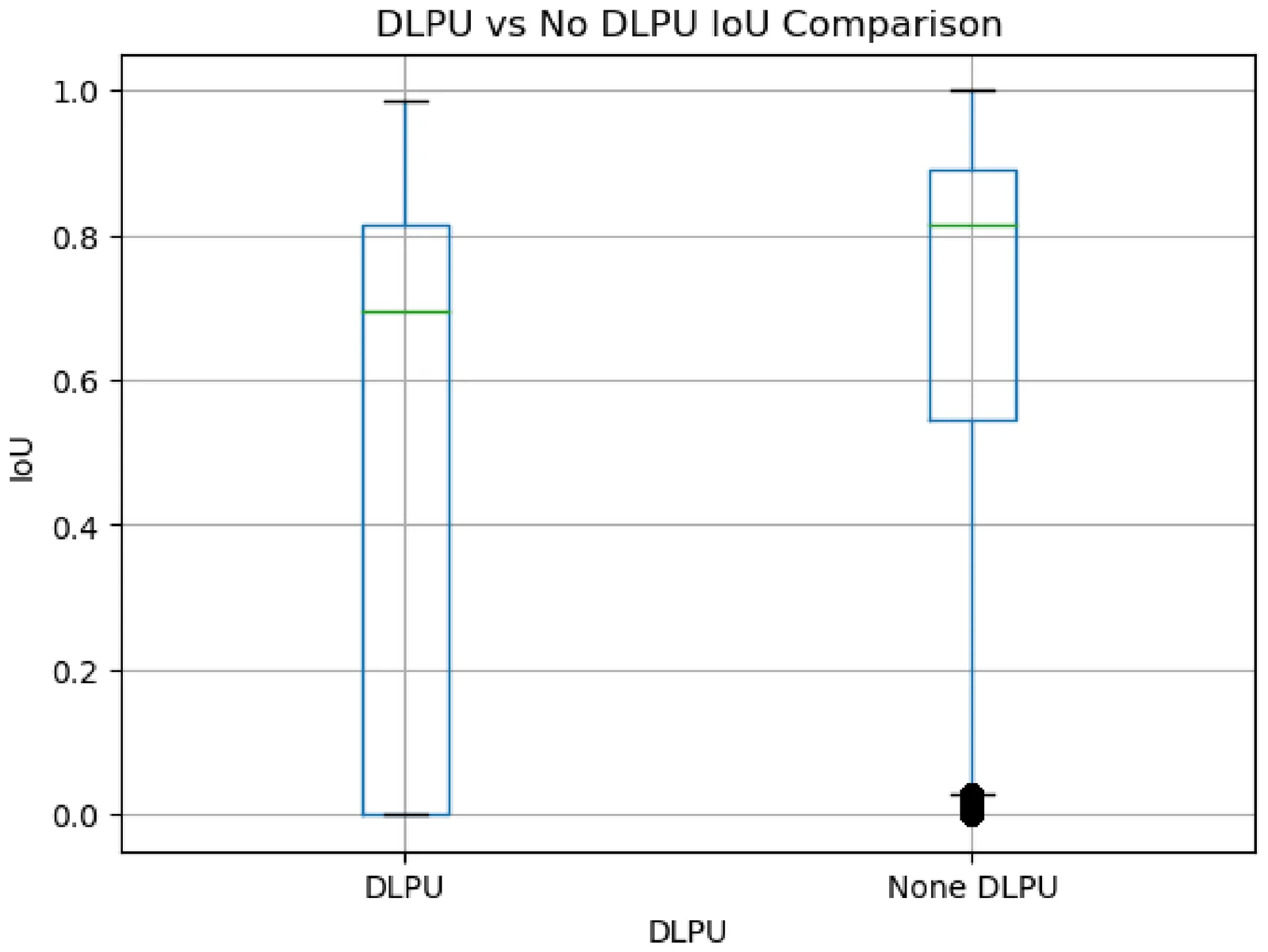
Comparison of IoU distributions grouped according to execution platform. The DLPU group contains the YOLOv5n INT8 configurations executed on the Axis ARTPEC-8, while the non-DLPU group contains all YOLOv5nu and YOLOv8n configurations executed on the Raspberry Pi 5, including FP32, FP16, dynamic-range, and INT8 models. The boxplots summarize differences in median IoU, interquartile variability, and lower-tail localization performance between the two execution environments.

The effect of hardware-accelerated inference (DLPU-based execution) on localization performance was evaluated by comparing the distribution of Intersection over Union (IoU) scores against non-DLPU Raspberry Pi 5 CPU configurations. The empirical distributions are presented in Figure 9, and the corresponding descriptive statistics are summarized in Table 7. The analysis focuses on central tendency, dispersion, and lower-tail behavior to assess robustness under hardware-constrained inference.

A pronounced structural difference is observable between the two execution paradigms. The non-DLPU configuration exhibits a higher median IoU and a relatively concentrated interquartile range, indicating stable and consistent bounding box regression performance. Although low-IoU outliers are present, they are comparatively sparse and confined to a narrow lower-bound region, suggesting strong robustness across heterogeneous input samples.

In contrast, the DLPU-based configuration demonstrates a substantially broader IoU distribution. While the upper whisker approaches near-perfect IoU values, confirming that high-accuracy detections remain attainable, the median IoU is noticeably reduced relative to the non-DLPU case. More importantly, the interquartile range expands significantly, and a considerable portion of the distribution extends toward near-zero IoU values. This heavy lower-tail behavior indicates increased susceptibility to severe localization degradation in certain samples.

The dispersion characteristics suggest that quantization and hardware-level execution constraints introduce additional variability into the regression process. Reduced numerical precision and limited dynamic range may amplify rounding errors, particularly in fine-grained spatial adjustments of bounding boxes. Consequently, although DLPU acceleration provides substantial gains in computational efficiency and energy consumption, it does so at the cost of reduced statistical stability in IoU performance.

From a robustness perspective, the non-DLPU configuration offers more predictable behavior, with tighter clustering around the median and reduced extreme-case variability. The DLPU setup, however, exhibits a bimodal tendency, where a subset of detections achieves high IoU values while another subset collapses toward minimal overlap. This phenomenon may reflect sensitivity to object scale, feature map discretization effects, or quantization-induced distortions in activation distributions.

Overall, the results demonstrate that hardware-accelerated inference significantly alters the IoU distribution profile. While peak performance remains comparable between DLPU and non-DLPU implementations, the increased variance and heavier lower tail observed in the DLPU case indicate reduced robustness under certain conditions. Therefore, deployment decisions involving DLPU acceleration should carefully balance computational efficiency gains against the potential impact on localization stability. Distribution-level evaluation proves essential in this context, as average IoU values alone would not adequately capture the observed variability and lower-bound degradation.

## 5. Discussion

The results demonstrate that system-level performance in edge AI pipelines is governed by the interaction between hardware, model architecture, and numerical precision, rather than any single factor. The experimental results reveal that the performance of edge-based object detection systems cannot be attributed to a single factor such as raw computational power or model architecture alone. Instead, system-level behavior emerges from the interaction between hardware capabilities, numerical precision, model design, and input resolution.

### 5.1. Hardware Acceleration Versus Architectural Flexibility

The comparison between the Axis ARTPEC-8 DLPU and the Raspberry Pi 5 CPU hig ights a fundamental trade-off. The DLPU provides a clear latency advantage at higher resolutions, achieving up to a 2.8× speedup at 640 × 640 input size. This confirms the effectiveness of dedicated accelerators for compute-intensive workloads.

However, this advantage is coupled with architectural constraints. The Axis platform requires strict INT8 quantization and supports a limited set of model structures, leading to measurable degradation in detection accuracy. In contrast, the Raspberry Pi platform allows for the use of more recent architectures, such as YOLOv5nu and YOLOv8n, which demonstrate improved robustness to quantization effects and consistently higher F1-scores.

These findings indicate that hardware acceleration alone does not guarantee superior system performance. The compatibility between model architecture and quantization strategy plays an equally important role. In this context, modern detector designs with decoupled heads appear inherently more resilient to reduced numerical precision.

### 5.2. Impact of Quantization on Robustness

The analysis of IoU distributions provides deeper insight into the effect of numerical precision beyond average performance metrics. While FP32, FP16, and dynamic quantization produce nearly identical central tendencies, INT8 quantization introduces a broader distribution with a significantly heavier lower tail.

This behavior suggests that quantization primarily affects robustness rather than peak performance. Although high-IoU detections remain achievable, the increased frequency of low-IoU outliers indicates a higher probability of failure in challenging scenarios, such as small object detection or complex backgrounds.

From a deployment perspective, this implies that evaluating only mean accuracy metrics (e.g., F1-score) is insufficient. Distributional characteristics, including variance and outlier density, must also be considered when assessing model reliability in real-world applications.

### 5.3. Hybrid-Resolution Optimization in Multi-Stage Pipelines

The results reveal an important system-level optimization opportunity that is specific to multi-stage detection pipelines. In the evaluated ALPR architecture, different stages operate on inputs with fundamentally different spatial characteristics, which enables the use of heterogeneous input resolutions within the same pipeline.

In the first stage, vehicle detection is performed on the full-resolution image, where objects occupy a relatively small portion of the frame. In this case, higher input resolutions (e.g., 640×640) are necessary to preserve sufficient spatial detail for reliable detection. In contrast, the second stage operates on cropped regions corresponding to detected vehicles. Within these regions, license plates occupy a significantly larger relative area, making it possible to reduce the input resolution without substantially degrading detection performance.

This observation suggests a hybrid-resolution processing strategy, where different stages of the pipeline operate at different input resolutions. For example, vehicle detection can be performed at 640×640, followed by license plate detection at 416×416 or 320×320. Such a configuration reduces the overall computational cost while preserving the spatial fidelity required for accurate detection in each stage.

From a system perspective, this approach provides a practical mechanism to balance latency and accuracy without modifying the underlying model architecture. The benefit is particularly relevant in DLPU-based systems, where multiple inference steps must be executed within a limited time budget. By selectively reducing the resolution of downstream stages, the total pipeline latency can be significantly reduced, enabling higher throughput or improved real-time performance.

More generally, the results indicate that resolution should not be treated as a global parameter in multi-stage pipelines. Instead, stage-specific resolution selection represents an additional optimization dimension that can complement model design and hardware acceleration strategies.

### 5.4. Resolution as a Dominant Design Parameter

Input resolution emerges as one of the most influential factors affecting detection performance. Increasing resolution consistently improves median IoU and F1-score values, confirming that higher spatial detail enhances localization accuracy.

However, the results also reveal a non-trivial trade-off. While 640 × 640 configurations achieve the highest peak accuracy, they exhibit increased variance and, in some cases, a bimodal performance distribution. This indicates that higher resolution does not uniformly improve robustness, but rather amplifies both successful and failure cases.

The 416 × 416 resolution appears to provide the most balanced configuration, offering a favorable compromise between accuracy, stability, and computational cost. This observation is particularly relevant for edge deployments, where resource constraints must be carefully managed.

### 5.5. DLPU-Induced Distributional Shift

A key observation of this study is the distributional shift introduced by DLPU-based inference. Compared to CPU execution, the DLPU configuration exhibits lower median IoU and significantly increased dispersion.

This effect can be attributed to the combined impact of fixed-point arithmetic, limited dynamic range, and hardware-specific execution constraints. While these factors enable substantial gains in efficiency, they also introduce additional variability in bounding box regression.

The resulting bimodal behavior—where some detections achieve high accuracy while others fail significantly—hig ights a critical limitation of hardware-accelerated inference. For applications requiring consistent performance, this variability must be explicitly addressed, potentially through quantization-aware training or model redesign.

### 5.6. System-Level Implications for Multi-Stage Pipelines

The evaluation of the Triple-DNN ALPR pipeline demonstrates that system-level optimization must consider the cumulative effect of multiple inference stages. Even moderate per-stage latency can lead to substantial total processing time when applied sequentially.

The results show that real-time operation (>1 FPS) is achievable on both platforms, but under different configurations. The Axis platform achieves this at full resolution due to hardware acceleration, while the Raspberry Pi requires reduced input resolution to meet the same constraint.

Importantly, the pipeline structure introduces an opportunity for resolution-aware optimization. Since license plate detection operates on cropped regions, lower resolutions can be used in later stages without significantly affecting detection quality. This hybrid-resolution strategy reduces total latency while preserving critical spatial detail where it is most needed.

### 5.7. Thermal and Energy Considerations

In addition to computational performance, energy efficiency and thermal behavior are important considerations in practical edge deployments. While this study does not include direct power measurements, the observed behavior is consistent with the architectural characteristics reported in the literature.

Dedicated AI accelerators, such as the DLPU used in the ARTPEC-8 platform, are specifically designed for low-power inference and typically operate with significantly lower energy consumption compared to general-purpose CPUs [8,10]. As a result, they enable sustained high-load operation under passive cooling conditions, which is particularly advantageous in compact and outdoor industrial environments.

In contrast, general-purpose platforms such as the Raspberry Pi 5 rely on CPU-based execution, where maintaining peak performance is often associated with increased power consumption and thermal load. This may require active cooling and can introduce performance variability under prolonged workloads.

Although these observations are not based on direct measurements in this study, they hig ight an important practical trade-off between flexibility and energy efficiency that should be considered when selecting deployment platforms for edge AI systems.

### 5.8. Engineering Implications

Overall, the findings emphasize that optimal edge AI system design requires a holistic approach. The best performance is achieved not by maximizing a single parameter, but by carefully balancing:

- Model architecture (robustness to quantization);
- Numerical precision (efficiency vs. stability);
- Input resolution (accuracy vs. latency);
- Hardware characteristics (acceleration vs. flexibility).

The results demonstrate that hybrid strategies—such as combining resolution scaling with architecture-aware model selection—offer a practical path toward achieving favorable speed–accuracy trade-offs in real-world edge deployments.

### 5.9. Limitations

Despite the comprehensive experimental evaluation, several limitations of the present study should be acknowledged.

First, the analysis was conducted using a single benchmark dataset (UA-DETRAC), which, although representative of real-world traffic scenarios, may not fully capture the diversity of deployment conditions encountered in practical ALPR systems. Variations in camera angle, resolution, environmental conditions, and regional license plate characteristics could influence system performance. Therefore, the generalizability of the results to other datasets and application domains should be interpreted with caution.

Second, the quantitative evaluation is restricted to the first-stage full-frame vehicle detector. The license plate detector and OCR component were not included in the reported latency, F1-score, or IoU measurements. Consequently, the presented results do not constitute an end-to-end evaluation of the complete ALPR pipeline. Although the first detector represents an important computational component, the cumulative latency and recognition accuracy of the complete system cannot be inferred directly from the present measurements. Future work will therefore include a dataset with license plate bounding boxes and transcriptions, stage-specific evaluation of the downstream components, and complete end-to-end latency and recognition measurements. While this assumption is justified from a performance perspective, the OCR stage was not quantitatively benchmarked. In real-world deployments, OCR accuracy and latency may introduce additional constraints that were not explicitly analyzed in this work.

Third, this study is limited to YOLO-based model architectures. Although these models provide a strong baseline for real-time object detection, alternative architectures (e.g., transformer-based detectors or lightweight non-YOLO models) may exhibit different behavior under quantization and hardware acceleration. Consequently, the conclusions regarding robustness and performance trade-offs may not directly extend to all model families.

Fourth, the hardware evaluation is restricted to a single generation of dedicated accelerator (Axis ARTPEC-8). While this platform is widely used in current industrial deployments, newer architectures (e.g., ARTPEC-9) may provide improved operator support, memory handling, and quantization strategies, potentially altering the observed trade-offs.

Fifth, although thermal and energy considerations are discussed from a system-level perspective, no direct power consumption measurements were performed. As a result, conclusions regarding energy efficiency are based on architectural characteristics and prior literature rather than empirical measurements.

Finally, the analysis relies on post-training quantization techniques without incorporating quantization-aware training. While this reflects common industrial deployment practices, more advanced training strategies could potentially mitigate the observed degradation in robustness, particularly for INT8 and DLPU-based configurations.

These limitations provide directions for future work, including multi-dataset evaluation, end-to-end pipeline benchmarking, exploration of alternative architectures, and hardware-aware training methodologies.

An important limitation of the cross-platform comparison is that identical neural network graphs could not be deployed in both execution environments. The Axis ARTPEC-8 results were obtained using YOLOv5n with the vendor-specific INT8 conversion workflow, whereas the Raspberry Pi 5 results include YOLOv5nu and YOLOv8n executed through TensorFlow Lite. Therefore, the reported cross-platform differences represent complete deployment-stack comparisons and should not be interpreted as a controlled hardware-only benchmark. A strict hardware comparison would require identical model weights, network structure, numerical precision, preprocessing, postprocessing, and decision thresholds on both platforms.

## 6. Conclusions

This work investigated the interaction between hardware acceleration, model architecture, and numerical precision in the context of a multi-stage edge AI pipeline for automatic license plate recognition.

The experimental results show that the evaluated Axis ARTPEC-8 deployment configuration achieved substantially lower vehicle-detector inference latency than the evaluated Raspberry Pi 5 configurations, particularly at higher input resolutions. Because the compared configurations differ in model structure, numerical representation, conversion workflow, and inference runtime, this result should be interpreted as a deployment-stack comparison rather than as an isolated measurement of hardware performance.

In contrast, general-purpose ARM platforms provide greater flexibility, enabling the use of more recent model architectures that are inherently more robust to quantization effects. While these platforms lack dedicated acceleration, the results show that resolution scaling can partially compensate for this limitation, allowing them to approach real-time performance under constrained settings.

A key finding of this study is that system performance is determined by the joint effect of multiple design choices rather than any single factor. In particular, the combination of model architecture and quantization strategy has a direct impact on robustness, while input resolution strongly influences both accuracy and latency. Hybrid-resolution processing pipelines were identified as an effective approach to balance these competing objectives, especially in multi-stage systems such as ALPR.

From a practical perspective, the results hig ight that edge AI system design must consider not only computational performance, but also thermal behavior and long-term operational stability. The passive cooling capability of the Axis platform makes it well suited for industrial deployments, whereas CPU-based solutions require more careful thermal management.

Overall, this study demonstrates that achieving an optimal speed–accuracy trade-off in embedded vision systems requires a holistic design approach. Future work may extend the analysis to newer hardware platforms, such as ARTPEC-9, investigate hardware-accelerated preprocessing pipelines, and explore detector architectures specifically optimized for fixed-point execution.

## Figures and Tables

**Table 1:** Comparison of the evaluated SoC platforms.

Parameter	Raspberry Pi 5 CPU	Axis Smart Camera (ARTPEC-8 SoC)
SoC	Broadcom BCM2712 [18]	Axis ARTPEC-8 [19]
CPU type	Cortex-A76 [20]	Cortex-A53 [21]
Number of cores	4	2
Max frequency	2.4 GHz	1.2 GHz
Acceleration	ARM NEON SIMD	Dedicated DLPU
CPU performance	10.7 DMIPS/MHz [20]	2.3 DMIPS/MHz [21]
Design focus	Performance-oriented	Efficiency-oriented

**Table 2:** Comparison of platform-specific software architectures.

Function	Axis Camera (ARTPEC-8)	Raspberry Pi (Reference)
Development environment	Native ACAP SDK (ANSI C)	Debian (C++17)
Inference engine	LAROD API (DLPU)	TensorFlow Lite (XNNPACK)
Acceleration	Dedicated DLPU (INT8)	CPU NEON (FP32/INT8)
Image processing	Low-level (stb_image)	High-level (OpenCV)
Input source	File system (JPG decoding)	File system (JPG decoding)
Preprocessing	Manual memory handling	Object-based (cv::Mat)
Postprocessing (NMS)	Custom C implementation	Built-in (OpenCV)

**Table 3:** Descriptive statistics of the IoU observations for all evaluated vehicle-detector configurations.

Model	Platform	Input Resolution	Precision	Mean IoU	SD	Min.	Q1	Median	Q3	Max.
YOLOv5nu	Raspberry Pi 5	320×320	Dynamic	0.719	0.277	0.000	0.703	0.822	0.885	1.000
YOLOv5nu	Raspberry Pi 5	320×320	FP16	0.720	0.277	0.000	0.699	0.823	0.887	1.000
YOLOv5nu	Raspberry Pi 5	320×320	FP32	0.720	0.277	0.000	0.698	0.823	0.887	1.000
YOLOv5nu	Raspberry Pi 5	320×320	INT8	0.719	0.277	0.000	0.703	0.822	0.885	1.000
YOLOv5n	Axis ARTPEC-8	320×320	INT8	0.571	0.315	0.000	0.431	0.705	0.805	0.976
YOLOv5nu	Raspberry Pi 5	416×416	Dynamic	0.680	0.327	0.000	0.655	0.824	0.891	1.000
YOLOv5nu	Raspberry Pi 5	416×416	FP16	0.681	0.328	0.000	0.655	0.826	0.892	1.000
YOLOv5nu	Raspberry Pi 5	416×416	FP32	0.680	0.328	0.000	0.654	0.825	0.892	1.000
YOLOv5nu	Raspberry Pi 5	416×416	INT8	0.680	0.327	0.000	0.655	0.824	0.891	1.000
YOLOv5n	Axis ARTPEC-8	416×416	INT8	0.549	0.343	0.000	0.062	0.708	0.817	0.980
YOLOv5nu	Raspberry Pi 5	640×640	Dynamic	0.600	0.383	0.000	0.009	0.810	0.891	1.000
YOLOv5nu	Raspberry Pi 5	640×640	FP16	0.600	0.384	0.000	0.008	0.810	0.891	1.000
YOLOv5nu	Raspberry Pi 5	640×640	FP32	0.599	0.384	0.000	0.007	0.811	0.891	1.000
YOLOv5nu	Raspberry Pi 5	640×640	INT8	0.600	0.383	0.000	0.009	0.810	0.891	1.000
YOLOv5n	Axis ARTPEC-8	640×640	INT8	0.483	0.378	0.000	0.000	0.672	0.818	0.985
YOLOv8n	Raspberry Pi 5	320×320	Dynamic	0.694	0.301	0.000	0.675	0.812	0.883	1.000
YOLOv8n	Raspberry Pi 5	320×320	FP16	0.691	0.305	0.000	0.669	0.812	0.884	1.000
YOLOv8n	Raspberry Pi 5	320×320	FP32	0.691	0.305	0.000	0.669	0.812	0.884	1.000
YOLOv8n	Raspberry Pi 5	320×320	INT8	0.694	0.301	0.000	0.675	0.812	0.883	1.000
YOLOv8n	Raspberry Pi 5	416×416	Dynamic	0.663	0.340	0.000	0.634	0.818	0.889	1.000
YOLOv8n	Raspberry Pi 5	416×416	FP16	0.659	0.344	0.000	0.624	0.818	0.890	1.000
YOLOv8n	Raspberry Pi 5	416×416	FP32	0.658	0.344	0.000	0.624	0.818	0.890	1.000
YOLOv8n	Raspberry Pi 5	416×416	INT8	0.663	0.340	0.000	0.634	0.818	0.889	1.000
YOLOv8n	Raspberry Pi 5	640×640	Dynamic	0.583	0.392	0.000	0.000	0.807	0.889	1.000
YOLOv8n	Raspberry Pi 5	640×640	FP16	0.580	0.394	0.000	0.000	0.805	0.888	1.000
YOLOv8n	Raspberry Pi 5	640×640	FP32	0.579	0.394	0.000	0.000	0.804	0.889	1.000
YOLOv8n	Raspberry Pi 5	640×640	INT8	0.583	0.392	0.000	0.000	0.807	0.889	1.000

Note: Q1 and Q3 denote the 25th and 75th percentiles, respectively; SD denotes the standard deviation. The Raspberry Pi 5 YOLOv5 rows correspond to the YOLOv5nu variant, whereas the Axis ARTPEC-8 rows correspond to YOLOv5n executed in INT8 precision on the DLPU.

**Table 4:** Descriptive statistics of the IoU observations grouped by YOLO model family.

Model Family	Mean IoU	SD	Min.	Q1	Median	Q3	Max.
YOLOv5-based	0.621	0.354	0.000	0.458	0.793	0.877	1.000
YOLOv8n	0.631	0.362	0.000	0.479	0.812	0.888	1.000

Note: Q1 and Q3 denote the 25th and 75th percentiles, respectively; SD denotes the standard deviation. The YOLOv5-based group combines the Raspberry Pi 5 YOLOv5nu configurations and the Axis ARTPEC-8 YOLOv5n INT8 configurations.

**Table 5:** Descriptive statistics of the IoU observations grouped by input resolution.

Input Resolution	Mean IoU	SD	Min.	Q1	Median	Q3	Max.
320×320	0.685	0.299	0.000	0.651	0.803	0.877	1.000
416×416	0.654	0.339	0.000	0.602	0.809	0.884	1.000
640×640	0.577	0.389	0.000	0.000	0.794	0.884	1.000

Note: Q1 and Q3 denote the 25th and 75th percentiles, respectively; SD denotes the standard deviation. Each row aggregates all evaluated model families, numerical precision formats, and execution environments at the corresponding input resolution.

**Table 6:** Descriptive statistics of the IoU observations grouped by numerical precision.

Numerical Precision	Mean IoU	SD	Min.	Q1	Median	Q3	Max.
Dynamic range	0.642	0.355	0.000	0.552	0.815	0.889	1.000
FP16	0.640	0.357	0.000	0.537	0.815	0.889	1.000
FP32	0.639	0.357	0.000	0.536	0.815	0.889	1.000
INT8	0.598	0.359	0.000	0.252	0.774	0.867	1.000

Note: Q1 and Q3 denote the 25th and 75th percentiles, respectively; SD denotes the standard deviation. The INT8 group contains both Raspberry Pi 5 and Axis ARTPEC-8 configurations, whereas the Dynamic, FP16, and FP32 groups contain Raspberry Pi 5 results only.

**Table 7:** Descriptive statistics of the IoU observations grouped by execution environment.

Execution Environment	Mean IoU	SD	Min.	Q1	Median	Q3	Max.
Axis ARTPEC-8 DLPU	0.526	0.354	0.000	0.000	0.696	0.814	0.985
Raspberry Pi 5 CPU	0.641	0.356	0.000	0.545	0.815	0.889	1.000

Note: Q1 and Q3 denote the 25th and 75th percentiles, respectively; SD denotes the standard deviation. The Axis group contains YOLOv5n INT8 configurations executed on the ARTPEC-8 DLPU. The Raspberry Pi group contains YOLOv5nu and YOLOv8n configurations evaluated using FP32, FP16, dynamic-range, and INT8 representations.

## Data Availability

The datasets presented in this article are not readily available due to institutional and data management restrictions. Requests to access the datasets should be directed to the corresponding author.

## Abbreviations

The following abbreviations are used in this manuscript:

ALPR	Automatic License Plate Recognition
DLPU	Deep Learning Processing Unit
NPU	Neural Processing Unit
TPU	Tensor Processing Unit
CPU	Central Processing Unit
GPU	Graphics Processing Unit
IoU	Intersection over Union
NMS	Non-Maximum Suppression
FP32	32-bit Floating Point
FP16	16-bit Floating Point
INT8	8-bit Integer Precision
PDP	Power-Delay Product
OCR	Optical Character Recognition
SIMD	Single Instruction Multiple Data
API	Application Programming Interface
SoC	System on Chip
ARM	Advanced RISC Machine
NEON	Advanced SIMD Extension for ARM
DNN	Deep Neural Network
FPS	Frames Per Second
